# A case of intestinal schwannoma initially suspected by transvaginal ultrasound

**DOI:** 10.1016/j.radcr.2022.08.028

**Published:** 2022-09-16

**Authors:** Laura López-Marín, Alejandro Olloqui, Ana Villalba, José Manuel Puente, Alberto Galindo

**Affiliations:** a Department of Obstetrics and Gynecology, Hospital Universitario 12 de Octubre, Avda. Córdoba s/n, Madrid 28041, Spain; b Department of Obstetrics and Gynecology, Hospital General de Villalba, Villalba 28400, Spain; c Fetal Medicine Unit, Department of Obstetrics and Gynecology, Hospital Universitario 12 de Octubre, Madrid 28041, Spain

**Keywords:** Diagnosis, Differential, Gastrointestinal stromal tumors, Gynecological examination, Schwannoma, Ultrasound

## Abstract

Schwannomas are peripheral nerve sheath tumors. Due to their low incidence, few cases of colorectal schwannomas have been published, which increases the diagnostic challenge. The aim of this case report is to discuss the role of transvaginal ultrasound in different areas than the gynecological disorders, when on hands of properly trained professionals that perform systematized procedures. A 56-year-old woman consulted for postmenopausal genital bleeding. During transvaginal ultrasound, a colonic solid, hypervascularized mass of 23 × 26 mm was visualized. As a result of this incidental finding, the patient underwent a sigmoidectomy, with a final diagnosis of intestinal schwannoma. Transvaginal ultrasound is today one of the most useful and accurate diagnostic tools in the assessment of gynecological disorders. However, the proximity of other pelvic structures makes it possible to evaluate the presence of nongynecological conditions. This fact should encourage gynecologists to systematize the transvaginal ultrasound procedure.

## Introduction

Mesenchymal neoplasms affecting the gastrointestinal tract are currently classified into 2 groups. Firstly, gastrointestinal stromal tumors (GISTs), and secondly, a smaller group that includes a wide spectrum of non-specific tumors, such as leiomyomas, sarcomas, lipomas and peripheral nerve sheath tumors [1]. Schwannomas are a subtype of peripheral nerve sheath tumors, since they originate from Schwann cells, and typically appear in extremities or in the central nervous system [2]. Gastrointestinal schwannomas constitute 2%-6% of all gastrointestinal mesenchymal tumors [3,4], with a reported incidence ratio between GIST and intestinal schwannoma of around 50-100 to 1 [5]. The most common location for schwannomas is the stomach, followed by the small intestine (83% and 12%, respectively). Colorectal schwannomas are rare, except for the microcystic/reticular variant that more commonly affects the large intestine [6].

They usually present at an average age of 60-65 years, and recent studies suggest they may affect women more frequently than men [7]. Most patients appear asymptomatic and when symptoms are present, they are normally nonspecific, such as abdominal pain or melena. Intestinal intussusception and obstructive disorders, although unusual, can also be found, and are directly related to the size and location of the tumor [8].

The final diagnosis of this kind of tumors is made by histopathological and immunohistochemical analysis (presence of S-100 protein and vimentin), and often requires surgical resection, as submucosal biopsy is commonly insufficient [9]. Colonoscopy, endoscopic ultrasonography (EUS) and abdominal computed tomography (CT) are useful techniques to characterize the lesion [10]. In fact, most colonic schwannomas are first identified in a screening colonoscopy [5,11]. The role of transvaginal ultrasound in this aspect has not been described in the literature.

## Case report

A 56-year-old female patient with no relevant past medical history, except for hyperthyroidism and a cesarean section, was referred to our Gynecology Unit because of postmenopausal uterine bleeding. A pelvic examination and an endometrial biopsy were carried out with no abnormal findings. Subsequently, a transvaginal ultrasound was performed, visualizing a thin endometrial line without other uterine or adnexal findings. The ultrasound equipment employed was a GE Voluson 730 PRO device (General Electric, Healthcare, Zipf, Austria). Cervical pathology screening was performed by colposcopy and cytology, both within normality. Afterwards, a hysteroscopy was undertaken, which showed an atrophic endometrium, with no findings of interest.

**Fig. 1:**
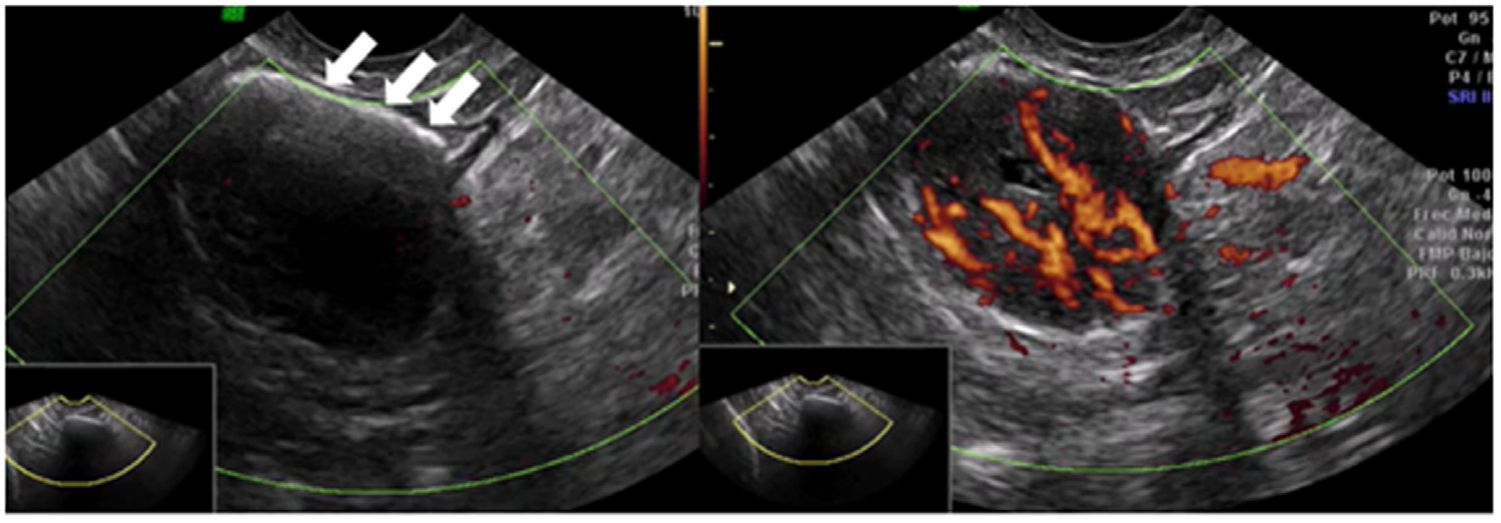
Intestinal schwannoma: transvaginal ultrasound. On the left, a hyperechogenic thin line (arrows) corresponding to intestinal gas can be seen. On the right, Power Doppler of the tumor, strongly vascularized. See also Videoclips 1 and 2.

Despite the absence of other episodes of metrorrhagia, a new follow-up transvaginal ultrasound was performed one year later. Once again, the endometrial midline appeared linear, both ovaries were atrophic, and no genital abnormalities were evident. Incidentally, a hypoechogenic nodular image was visualized in the colonic lumen, sized 26 × 23 mm and surrounded by an hyperechogenic content congruent with intestinal gas. It presented strong vascularization (vascular score 4/4) and was located more than 18 cm distal to the anal verge, in a retrovesical location (Fig. 1 and Videoclips 1 & 2).

After consultation with the Digestive Tract Department, a colonoscopy was performed. Biopsies were taken from an inflammatory-like submucous lesion found 30 cm from the anal verge. Histopathological report described a mesenchymal subepithelial tissue with neural immunophenotype and low proliferation score, suggesting schwannoma or neurofibroma as first diagnostic possibilities. Because of the shallowness of the resection achievable by colonoscopy and/or echoendoscope, the patient underwent laparoscopic sigmoidectomy with latero-lateral primary anastomosis. Histopathological examination of the surgical specimen confirmed the diagnosis of a fusiform-type schwannoma. The postoperative evolution was uneventful and the last follow-up was assessed at 6 months, and it was also normal.

## Discussion

Transvaginal ultrasound is today one of the most accurate and commonly used diagnostic tools in the assessment of female genital tract disorders, given the improvement both in ultrasound equipment and professional training. The diagnosis of extragenital pathology is not a primary objective of standard transvaginal ultrasound, although it is commonly used to quantify the extension of gynecological diseases outside the inner genital tract, such as endometriosis. The proximity of the rectum, colon, bladder, ureters, and pelvic vessels to the lower genital tract makes it feasible to theoretically visualize and diagnose pathology at each of these levels during the gynecological ultrasound examination.

In our case, the presence of a pelvic mass with a strong vascular component and solid character lead us to a differential diagnosis including pedunculated myomas and solid ovarian tumors. The continuity of the tumor with the sigmoid colon wall and the presence of a surrounding, thin hyperechoic lamina corresponding to intraluminal gas (Videoclips 1 and 2) gave us the diagnostic key, suggesting that the origin of the mass was intestinal. Further studies allowed us to classify it as an intestinal schwannoma that otherwise would have not been diagnosed. To the best of our knowledge, this is the first case of an intestinal schwannoma initially suspected by transvaginal ultrasound.

Although not yet systematized, transvaginal ultrasound is a useful technique to diagnose non-gynecological alterations located in the pelvis. Therefore, from our point of view, gynecologists should be encouraged to carry out a complete pelvic examination during transvaginal ultrasound, instead of focusing exclusively on the genital tract. They should be aware of the possible extra-gynecological findings that might come up during this procedure, so that an adequate assessment of the patient can be completed.

## Patient consent

Informed consent for publication was obtained from the subject of this case report, according to the World Medical Association Declaration of Helsinki, revised in 2000, Edinburgh.

## Appendix Supplementary materials

Image, video 1

Image, video 2
